# Investigating the Effects of Iron Dilution on the Corrosion Resistance of Inconel 625 Welding Overlay on Carbon Steel

**DOI:** 10.3390/ma18245574

**Published:** 2025-12-11

**Authors:** Alexandre Borghi Cunha, Jan Vatavuk, Carlos Roberto Camello Lima

**Affiliations:** School of Engineering, Mackenzie Presbyterian University, Rua da Consolação 930, Higienópolis, São Paulo 01302-907, SP, Brazil; alexandre.borghi@hotmail.com (A.B.C.); jan.vatavuk@ifsp.edu.br (J.V.)

**Keywords:** Inconel 625, welding overlay, corrosion, clad plates, dilution

## Abstract

**Highlights:**

**What are the main findings?**

- Inconel 625 overlays exhibited refined dendritic microstructures.
- Iron dilution modified the distribution of Ni, Cr, Mo, and Nb.
- Pitting corrosion occurred only at ~19% Fe.
- Low-Fe overlays matched ERNiCrMo-3 composition and showed high integrity.

**What are the implications of the main findings?**

- Multiple buttering layers effectively reduce Fe dilution.
- A ~19% Fe limit is conservative under ASTM G48 Method A conditions.
- Optimized GMAW overlays improve corrosion resistance offshore.
- Results support relaxed Fe limits for cost-efficient cladding design.

**Abstract:**

This study investigates the influence of iron dilution on the microstructure and corrosion behavior of Inconel 625 weld overlays deposited on carbon steel. Different deposition strategies were employed to control dilution and to evaluate its effect on elemental segregation. The overlays were characterized in terms of microstructural evolution, chemical distribution, and corrosion performance under standardized testing conditions. The results show that increasing iron dilution enhances chemical segregation within the dendritic structure, which governs the initiation of localized corrosion. A critical dilution condition associated with the onset of pitting was identified. These findings advance the understanding of dilution-controlled corrosion mechanisms in nickel-based alloy overlays.

## 1. Introduction

With advances in offshore oil and natural gas exploration, it has become increasingly necessary to develop alloys capable of withstanding the severe conditions involved, offering high mechanical strength and resistance to corrosion and oxidation [1,2,3]. The discovery of deep-water oil reserves, such as the Brazilian Pre salt, has brought about the need for materials capable of operating in harsh environments, with water depths of up to 3000 m and salt and sediment layers reaching depths of up to 5000 m [4,5].

Exposure to production fluids requires corrosion resistant alloys due to the presence of corrosive agents such as dissolved H_2_S and CO_2_ in water (H_2_O). The main technological challenge is to make oil extraction viable under these conditions, especially using corrosion resistant materials [1,6,7,8].

Ni-based superalloys are characterized by a nickel-rich austenitic face-centered cubic (FCC) γ matrix, which provides excellent mechanical and corrosion resistance. Their properties result from the addition of alloying elements such as Cr, Fe, Co, Nb, and Mo, which mainly promote solid-solution strengthening by distorting the crystal lattice and hindering dislocation motion [9,10]. In precipitation-hardened Ni-based superalloys, strengthening is associated with γ′ (Ni_3_Al or Ni_3_Ti) and, in some systems, γ″ (Ni_3_Nb) phases. However, Inconel 625 is primarily a solid-solution-strengthened alloy, in which Nb and Mo play a key role in improving mechanical strength and corrosion resistance [9].

Ni-based alloys are the most studied materials for such applications because of their excellent corrosion resistance, high temperature strength, and oxidation resistance above 500 °C [11,12,13]. The presence of intermetallic phases and carbide precipitates in this alloy enhances solid-solution strengthening and can directly affect its weldability, particularly within the temperature range of 500–750 °C. In addition, the nucleation of secondary phases and precipitates may occur [14,15]. The presence of molybdenum and nickel provides Inconel 625 with excellent resistance to non-oxidizing corrosion environments, while chromium and nickel ensure protection against oxidizing corrosion [16]. One limitation to the widespread use of these alloys is the high cost of nickel compared to carbon steel, which makes the use of solid components economically unfeasible. However, nickel alloys offer better equipment integrity, increased service life, improved quality, and reduced repair frequency [1,17].

To make the use of nickel-based alloys economically viable, cladding has become one of the most widely used techniques. In this method, a lower cost base metal provides the mechanical properties, while a thin layer of expensive nickel alloy (up to 80 times more costly) is applied to provide corrosion resistance. This makes it a cost-effective option for using such materials [18,19].

Several welding processes are used for applying overlays. The selection of the most appropriate process depends on various factors such as base metal type, geometry, cost effectiveness, and consumable material cost [11,18,20].

Among the welding processes, Gas Metal Arc Welding (GMAW) stands out for its high productivity and relatively low cost compared to other techniques like Electroslag Welding. It also produces a heat affected zone (HAZ) that is less detrimental to the overlay, avoiding excessive hardness increases. For the overlay to be suitable for corrosion resistant applications, its chemical characteristics must match those of the consumable wire used [21,22,23]. Compared with other processes such as Gas Tungsten Arc Welding (GTAW), which typically results in lower dilution, the GMAW process is the most extensively studied due to its higher deposition rate. Moreover, dilution in GMAW can be effectively controlled by adjusting other welding parameters and process variables [7,15,24].

**Figure 1:**
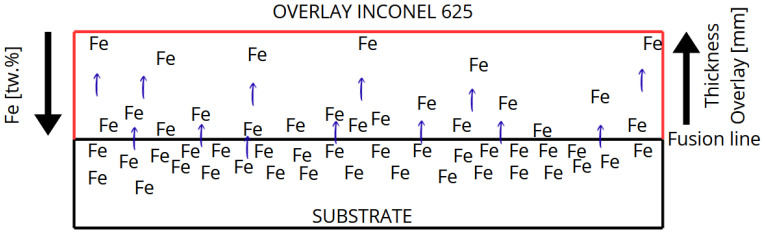
Schematic illustration of iron dilution from the carbon steel substrate into the Inconel 625 weld overlay during GMAW deposition, showing the Fe gradient from the fusion line to the top surface.

Due to dilution inherent in welding, the initial overlay layers incorporate elements from the base metal such as iron, as shown in Figure 1, which is highly prone to oxidation. A transition zone, known as the partially mixed zone, forms between the base metal and the overlay [19,25,26]. The dilution rate in the weld zone is also a key factor. When using high dilution processes, the altered chemical composition of the weld can significantly affect the overlay’s corrosion resistance [3].

Technical standards for chemical analysis acceptance criteria, such as API 582 (Welding Guidelines for the Chemical, Oil, and Gas Industries) and Petrobras Standard N-1707, set limits for iron content in the final layers of weld overlays. Because dilution is intrinsic to the welding process, controlling iron content in the final layer becomes more challenging when thin overlays up to 3 mm thick are required [26,27].

This study evaluates the corrosion resistance of Inconel 625 overlays with different iron contents in the final layer, deposited by GMAW on carbon steel plates.

Several studies have demonstrated that the iron content in Inconel 625 and Ni–Cr–Mo-based alloys plays a critical role in microstructural evolution, secondary phase formation, and localized corrosion resistance in chloride-containing environments. Lourenço et al. [28] investigated as-cast modified Inconel 625 alloys with Fe contents up to 15 wt.% and showed that Fe significantly affects Laves-phase morphology, carbide formation, and corrosion behavior in NaCl solution. DuPont [20] demonstrated that iron enrichment during weld solidification promotes the formation of Laves-type TCP phases, which consume Nb and Mo from the matrix and impair the stability of the passive film. More recently, Liu et al. [29] reported that in laser-cladded Ni–Cr–Mo–xFe coatings (0–25 wt.% Fe), alloys containing up to 5 wt.% Fe exhibit excellent resistance to pitting corrosion, whereas Fe contents in the range of 10–25 wt.% lead to metastable or stable pitting behavior in ferric chloride and simulated seawater environments, with pit initiation preferentially occurring in Mo-depleted dendritic regions.

**Figure 2:**
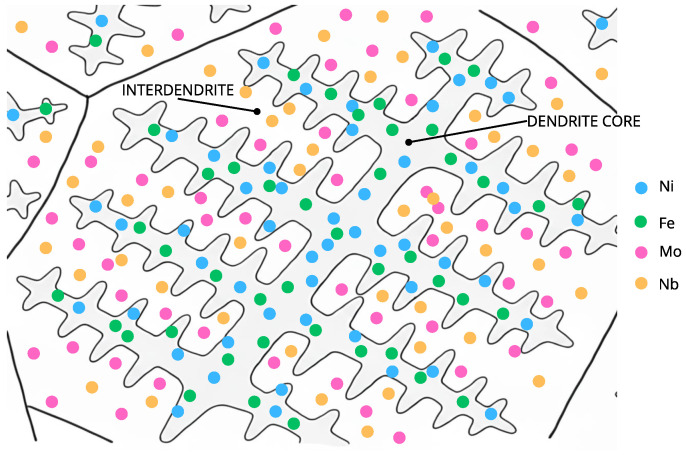
Schematic representation of microsegregation during solidification of the Inconel 625 weld overlay, showing preferential Nb and Mo enrichment in the interdendritic regions and Fe and Ni enrichment in the dendrite cores.

As shown in Figure 2, during the solidification of the Inconel 625 weld overlay, Nb and Mo preferentially segregate to the interdendritic regions, while Fe and Ni remain more concentrated in the dendrite cores. This elemental partitioning promotes the formation of Laves-type intermetallic phases in the interdendritic zones and locally reduces the stability of the passive film, increasing the susceptibility to localized corrosion [20,28,29].

From an industrial perspective, acceptance criteria such as ASTM G48 [30] and NORSOK M-601 [31] are widely adopted for the qualification of corrosion-resistant weld overlays for offshore and petrochemical applications. These standards are commonly associated with strict limits on Fe dilution in Inconel 625 overlays, typically recommending maximum Fe contents of about 3–5 wt.% for severe service and up to 10–15 wt.% for less aggressive environments. Functionally graded and Ni-based structures evaluated according to ASTM G48 have also been reported in the literature, as shown by Senthil et al. [32], reinforcing the relevance of ferric chloride testing for qualification of corrosion-resistant overlays. However, the applicability of these Fe limits depends strongly on the welding process, dilution control strategy, and the resulting microstructural condition. In this context, a systematic evaluation of the relationship between iron dilution, microsegregation, secondary phase formation, and compliance with ASTM G48 and NORSOK M-601 acceptance criteria remains necessary.

## 2. Materials and Methods

The base metal was selected in accordance with ASME Section II, Part A–SA-516 Grade 70, in the form of plates with dimensions of 12.5 mm × 200 mm × 250 mm, with chemical composition given in Table 1. This material was chosen due to its widespread industrial use in the manufacture of equipment for refineries and oil extraction platforms, such as heat exchangers, towers, and pressure vessels [33].

The filler metal was selected based on ASME Section II, Part C–SFA 5.14, ERNiCrMo-3, with chemical composition presented in Table 1. This filler metal exhibits a chemical composition similar to that of solid Inconel 625 [34], aiming to obtain a final weld overlay composition close to that of the consumable.

The shielding gas used was argon, in accordance with ASME Section II, Part C–SFA 5.32 SG-A [34]. The welding equipment used was a Lincoln Ideal Arc CV 400 power source (Lincoln Electric, Cleveland, OH, USA), with an LF-33 wire feeder. The carbon steel plates were prepared for welding by sand blasting.

**Figure 3:**
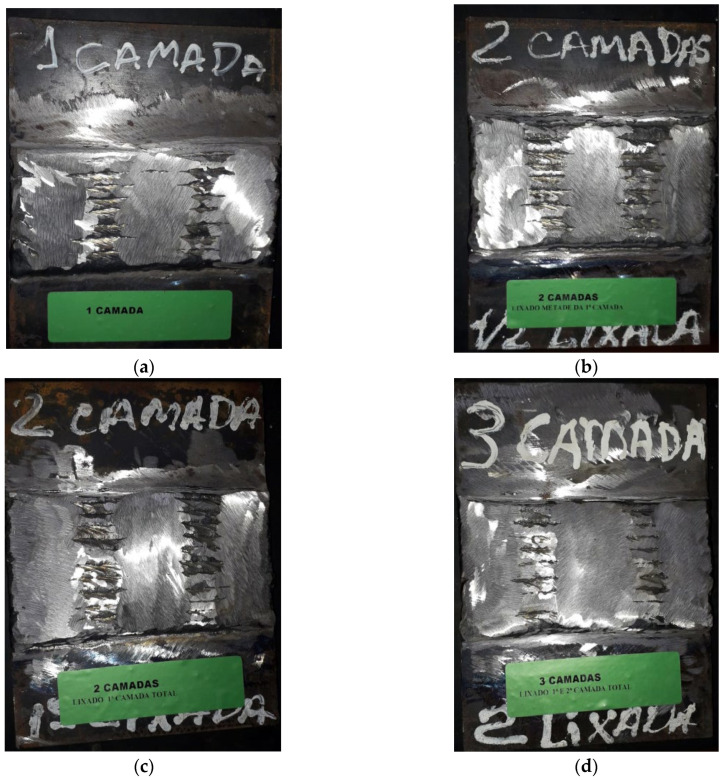
Welded overlays of the test specimens: (**a**) CP1, (**b**) CP2, (**c**) CP3, and (**d**) CP4.

The welding parameters used in this study are listed in Table 2. Figure 3a–d shows the welded test specimens produced in accordance with the parameters listed in Table 2 and the different bead deposition strategies described below.

**Figure 4:**
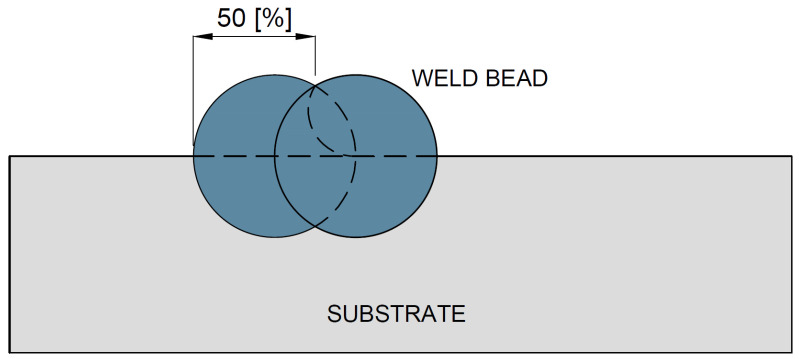
Schematic representation of the overlapping of weld beads for specimen CP1.

Each weld bead was mechanically ground to approximately 50% of its width in order to ensure adequate overlap of the subsequent beads, resulting in a total overlay width ranging from 70 mm to 90 mm, as schematically illustrated in Figure 4.

Variations in the welding procedure were employed to obtain different iron contents at the overlay surface, while keeping all welding parameters constant in order to avoid changes in the fused zone morphology and microstructure. The thickness of each deposited layer and the amount of material removed were controlled by mechanical measurements, based on the nominal layer thickness obtained from the welding parameters and verified by direct thickness measurements and cross-sectional references after grinding.

**Figure 5:**
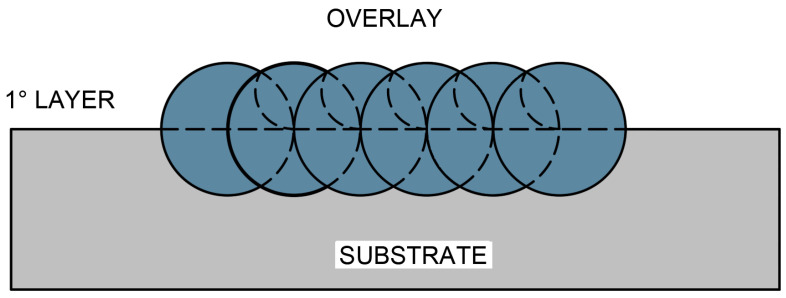
Welded overlay of test specimen CP1 produced with a single deposition layer, without subsequent material removal.

The first test specimen (CP1) was produced using a single overlay layer, without any subsequent material removals showed in Figure 5.

**Figure 6:**
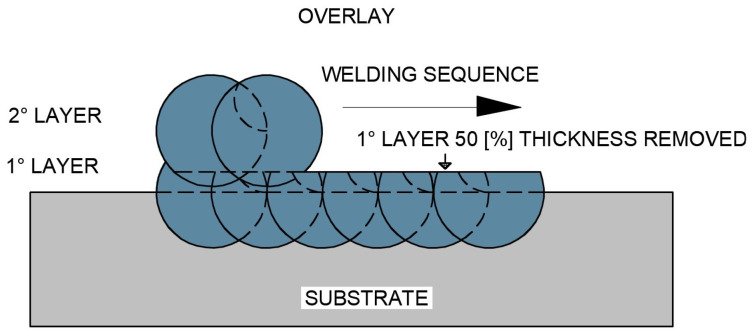
Schematic representation of the overlap of the first overlay layer after partial removal of approximately 50% of its thickness, followed by the deposition of the second overlay layer (CP2 configuration).

The second test specimen (CP2), schematically illustrated in Figure 6, was produced by depositing a complete first overlay layer, from which approximately 50% of its thickness was removed by controlled mechanical grinding, with the removed depth being monitored by comparing the initial deposited thickness with the remaining thickness after machining. Subsequently, a second overlay layer was deposited on top of the remaining material.

**Figure 7:**
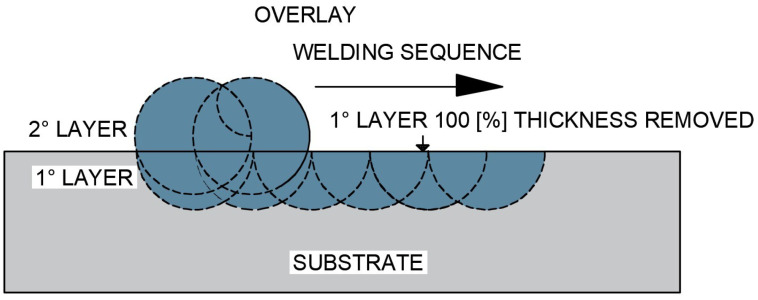
Schematic representation of the overlap of one overlay layer by welding after complete removal of the first deposited layer (CP3 configuration).

The third test specimen (CP3), as shown in Figure 7, was produced with a complete single overlay layer, whose entire thickness was removed by grinding until the surface became flush with the base metal, this condition being confirmed by thickness measurements and visual inspection of the weld interface. After complete removal of the first layer, a second overlay layer was deposited.

**Figure 8:**
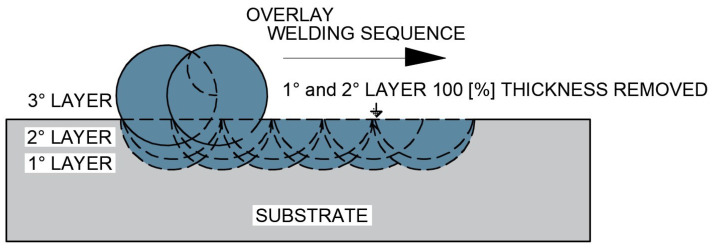
Schematic representation of the overlap of two overlay layers by welding after complete removal of the first and second deposited layers (CP4 configuration).

The fourth test specimen (CP4), schematically presented in Figure 8, was initially produced with a single overlay layer, which was completely removed under the same controlled grinding procedure. A second overlay layer was then deposited and entirely removed again. Finally, a third overlay layer was deposited to obtain the final surface condition.

**Figure 9:**
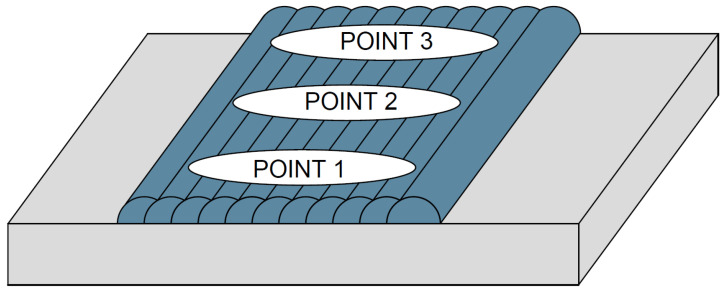
Locations of chemical analysis points.

Chemical analysis was performed using a portable X-ray spectrometer (Model: X-MET 5100, Oxford Instruments, Abingdon, UK) operated with Niton XL2 software (version 8.x, Thermo Fisher Scientific, Waltham, MA, USA), which measured the iron content on the final surface of each specimen. The surface was prepared by sanding with a 600-grit silicon carbide disc (4.25 in diameter) using a rotary sander, to ensure flatness and remove oxide scales from the weld passes. Three measurement points were analyzed on each specimen: at the start, middle, and end of the overlay end and recording the average, as shown in Figure 9.

The Specimens for microstructural analysis after welding were sectioned and sanded using a sequence of #220, #320, #400, #600, and #1200 grit papers, followed by final polishing with 0.25 µm diamond paste. The etchant used for attacking the weld metal was aqua regia, prepared with a 3:1 molar ratio of HCl to HNO_3_, applied by immersion for 30 s. Microstructural characterization was performed using an Olympus BX60 optical microscope (Olympus Corporation, Tokyo, Japan), equipped with an image acquisition system.

Corrosion testing was performed according to ASTM G-48, Method A, with standard NORSOK M-601 as the acceptance criterion. The test conditions were 50 °C and 24 h of immersion in ferric chloride. After testing, the overlay surfaces were first examined using an Olympus BX60 optical microscope (Olympus Corporation, Tokyo, Japan) and then analyzed by SEM using a HITACHI TM3000 (Hitachi High-Tech Corporation, Tokyo, Japan).

## 3. Results

### 3.1. Weld Integrity

**Figure 10:**
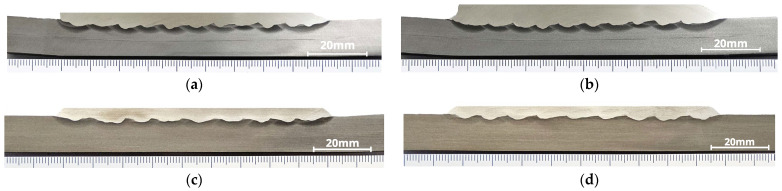
Transverse cross-section macrographs of the welded overlays: (**a**) CP1, (**b**) CP2, (**c**) CP3, and (**d**) CP4.

The welded specimens analyzed in this study did not exhibit any discontinuities such as porosity, cracks, or lack of fusion. All overlays were examined by liquid penetrant testing and metallographic cross-sectional analysis, as shown in Figure 10a–d, confirming complete fusion between the deposited layer and the substrate.

### 3.2. Chemical Analysis

#### 3.2.1. Composition Results

Table 3 presents the chemical composition values obtained for the welded overlays and their deviations relative to the nominal composition of the ERNiCrMo-3 filler metal. The chemical compositions were measured by X-ray fluorescence (XRF), and all results are reported in weight percentage wt.%.

Specimen 1 (CP1) showed a significant increase in Fe content and decrease in Ni, Cr, and Mo, resulting from high dilution near the fusion line.

Specimen 2 (CP2), which was welded with two layers removing 50% of the first before depositing the second layer the composition was near to the wire.

Specimen 3 (CP3), produced with a buttering layer, still presented moderate Fe dilution.

Specimen 4 (CP4), made with two buttering layers followed by a third overlay, exhibited minimal dilution and a composition almost identical to the ERNiCrMo-3 wire.

#### 3.2.2. Summary of Observations

- CP1 presented the highest Fe content ~19 wt.% and greatest deviation from nominal composition;
- CP2 and CP3 showed intermediate dilution effects;
- CP4 demonstrated the lowest Fe content and composition closest to the filler wire.

### 3.3. Microstructural Analysis

#### 3.3.1. Morphological Features

The overlays displayed both columnar and equiaxed dendritic structures typical of Inconel 625 weld overlays.

The secondary dendrite arm spacing was approximately 10 µm, consistent with high cooling rates and solute redistribution.

**Figure 11:**
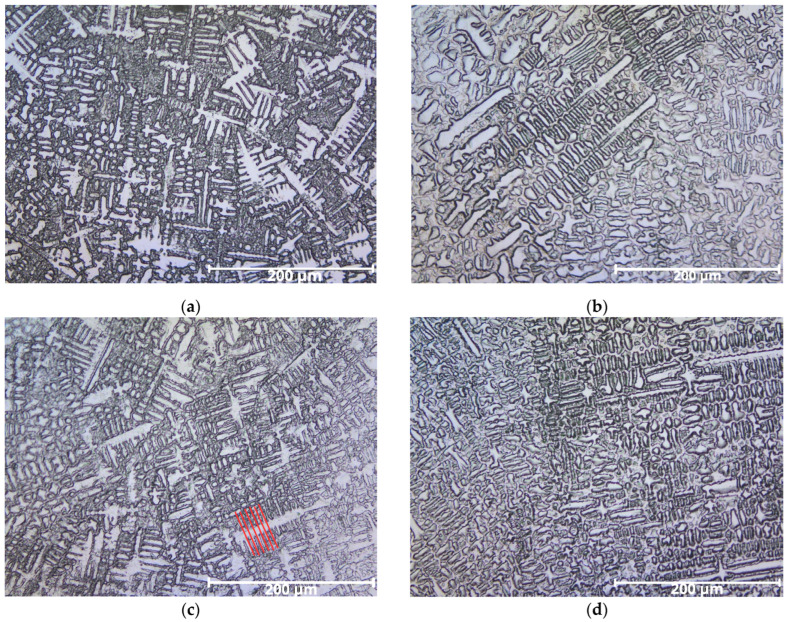
Micrographs of welded overlays showing columnar and equiaxed dendritic structures: (**a**) CP1, with higher dilution, presenting finer dendrites and more evident interdendritic segregation; (**b**) CP2, with reduced dilution, showing dendrites closer in composition to the filler metal; (**c**) CP3, with partial buttering, exhibiting intermediate microstructure and segregation; and (**d**) CP4, with multiple buttering layers, revealing dendritic morphology most similar to the ERNiCrMo-3 wire, with minimized iron contamination.

These microstructures are illustrated in Figure 11a–d.

#### 3.3.2. Comparative Analysis of Specimens

- CP1 (high dilution): finer dendrites and more evident interdendritic segregation;
- CP2 (moderate dilution): dendrites closer in composition to filler metal;
- CP3 (buttered): intermediate morphology;
- CP4 (multiple buttering layers): dendritic pattern similar to ERNiCrMo-3 wire with minimal Fe contamination.

### 3.4. Corrosion Testing and Pit Detection

#### 3.4.1. Test Description

Corrosion resistance was evaluated according to ASTM G48 Method A using ferric chloride solution to accelerate localized attack. After testing, all overlays were visually inspected for pitting.

#### 3.4.2. Pit Detection Results

**Figure 12:**
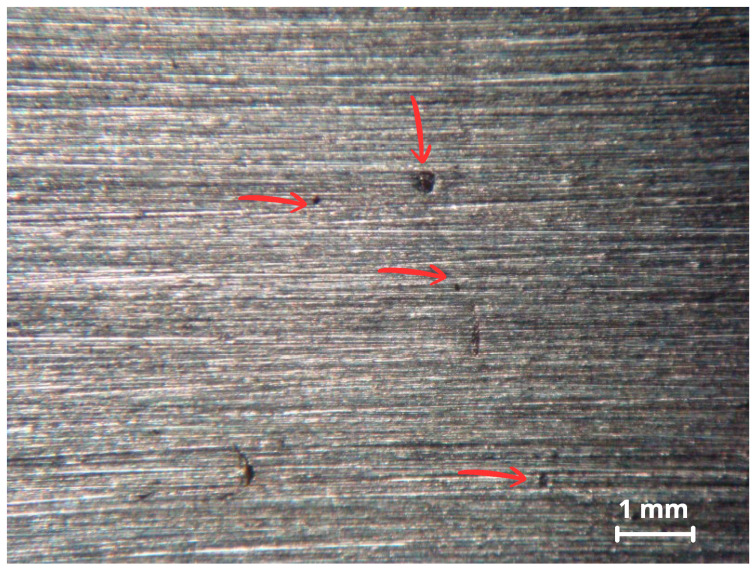
Surface analysis of CP1 via stereomicroscope showing some pits as indicated by the red arrows.

**Figure 13:**
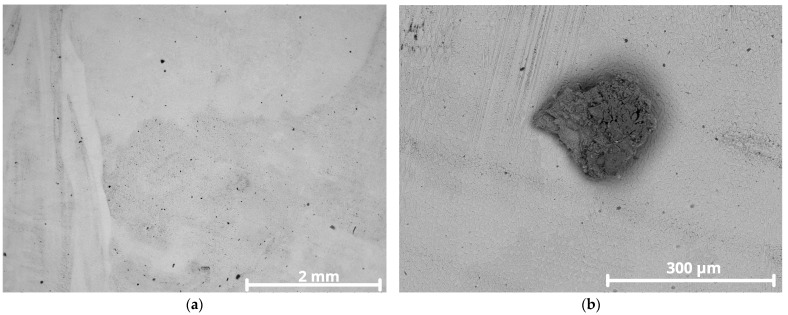
SEM images of CP1 overlay surface after corrosion testing: (**a**) pits distributed on the surface; (**b**) higher magnification of pit showing shallow depth.

Only specimen CP1 exhibited isolated pits, observed initially by stereomicroscope in Figure 12 and later confirmed by SEM Figure 13a,b.

The pits showed shallow morphology with low conductivity corrosion products. Specimens CP2, CP3, and CP4 presented no visible pitting under the same conditions.

### 3.5. Metallurgical Characterization of the Corroded Region

#### 3.5.1. Secondary Phase Identification

**Figure 14:**
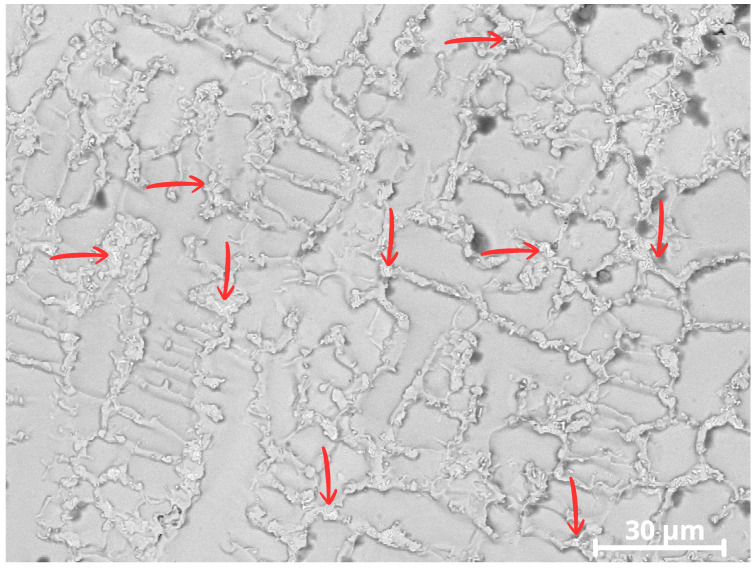
Image of CP1 showing secondary precipitates in the interdendritic regions. The red arrows indicate Laves-type intermetallic precipitates formed in the interdendritic zones.

Microstructural analyses by SEM with EDS revealed the presence of bright precipitates in the interdendritic regions of CP1, as seen in Figure 14, consistent with Laves-type (Fe,Ni)_2_(Nb/Mo) intermetallic phases showed by red arrow.

#### 3.5.2. Elemental Segregation

The EDS results showed segregation behavior of Fe, Cr, Ni, Nb, and Mo within the dendritic microstructure.

Partition coefficients

(1) $$K=\frac{C_{s}}{C_{l}}$$

were calculated and are summarized in Table 4.

#### 3.5.3. Chemical Mapping

**Figure 15:**
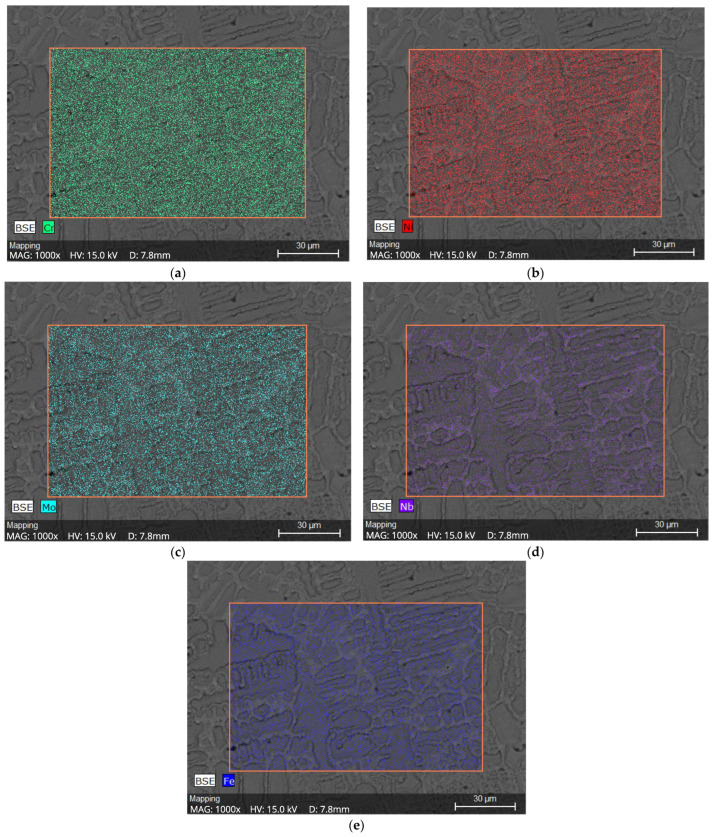
Chemical Mapping of CP1, (**a**) Cr distribution, (**b**) Ni distribution, (**c**) Mo distribution, (**d**) Nb distribution and (**e**) Fe distribution.

The color-coded maps in Figure 15a–e confirm that Mo and Nb were concentrated in interdendritic regions, while Fe and Ni were preferentially located in dendrite cores, consistent with the segregation behavior expected for Ni-based superalloys.

## 4. Discussion

The present results allow a direct comparison with previously reported limits for iron dilution in Inconel 625 and Ni–Cr–Mo-based overlays subjected to chloride-containing environments and qualification tests. In laser-cladded Ni–Cr–Mo–xFe coatings, Liu et al. [29] reported excellent pitting resistance for Fe contents up to 5 wt.%, whereas alloys containing 10–25 wt.% Fe exhibited metastable or stable pitting behavior, with selective attack occurring in Mo-depleted dendritic regions. Likewise, Lourenço et al. [28] showed that Fe contents in the range of 10–15 wt.% strongly modify Laves-phase morphology and microstructural features in modified Inconel 625, with a direct impact on corrosion behavior. DuPont [20] further demonstrated that increasing Fe content during solidification favors the formation of Laves-type phases, leading to Nb and Mo depletion in the matrix and a consequent reduction in localized corrosion resistance.

Table 3 presents the chemical composition values found and their deviations compared to the nominal composition of the ERNiCrMo-3 alloy wire. Solidification in nickel-based alloys typically results in dendritic microstructures. Inconel 625 overlays may exhibit both columnar and equiaxed dendritic structures [35,36]. During solidification, the chemical composition differs between interdendritic regions and dendrite cores due to microscale segregation of alloying elements [11,35,37,38,39]. However, since X-ray spectrometry analyzes a relatively large area, only average values are obtained.

Elements like Nb, Mo, Si, and C tend to segregate into the liquid phase during solidification, enriching the interdendritic regions, while elements like Fe, Cr, Co, and W segregate less and remain more uniformly distributed in the dendrite cores [39,40,41]. Research has obtained similar results using elemental chemical mapping via SEM and EDS, confirming the segregation behavior of alloying elements within the dendritic structure [41].

According to the tests performed, Specimen 1 (CP1) showed significant differences in chemical composition compared to the ERNiCrMo-3 wire. This is due to the high dilution caused by the proximity to the fusion line only 1.5 mm away. The most significantly altered elements were Fe and Ni, with Fe being the primary element in carbon steel (over 98 wt.%) and Ni being the major element in the ERNiCrMo-3 wire (>58 wt.%). Since Fe is highly soluble in Ni, it dissolves into the weld pool during dilution, leading to marked differences in surface chemistry.

For Specimen 2 (CP2), which was welded with two layers removing 50% of the first before depositing the second layer the composition was closer to that of the wire. This is because most of the dilution from the base metal occurred in the first layer, meaning the second layer had less Fe contamination and more closely resembled the filler metal.

In Specimen 3 (CP3), a buttering technique was used, where the initial layer was ground down flush with the substrate before depositing the second. This process reduced the iron content in the overlay surface due to minimized dilution from the carbon steel. As listed in Table 3, even with buttering, there was still notable dilution, particularly in Fe content, which remains elevated compared to the wire.

Specimen 4 (CP4) was produced aiming for an optimal overlay. Two buttering layers were applied and removed before depositing a third layer. This resulted in a surface with lower Fe content and chemical composition closely matching the ERNiCrMo-3 wire. The comparison among the four specimens (CP1–CP4) reveals significant differences in chemical composition resulting from dilution. CP1 exhibited the largest deviations from the ERNiCrMo-3 consumable, with Cr, Nb, and Mo contents below the specified range and excess Fe, reflecting the strong influence of dilution in the layer close to the fusion line.

CP2, although still showing Fe levels above the ideal, presented a composition closer to the wire, with adequate levels of Cr, Nb, and Mo, since the second layer reduced contamination from the substrate. CP3 showed an intermediate condition, with borderline Cr and Mo contents and Nb still below the recommended range, indicating that even with the buttering technique, dilution was not completely eliminated. Finally, CP4 displayed a composition most similar to the filler wire, with low Fe content and Cr, Nb, and Mo within the specified ranges, confirming that multiple buttering layers are effective in minimizing the influence of the carbon steel substrate on the overlay.

The overlays’ microstructures are shown in Figure 11a–d. They display both columnar and equiaxed dendritic structures typical of Inconel 625 weld overlays [34]. The secondary dendrite arm spacing marked with red lines in Figure 11c, is small, around 10 µm due to solute redistribution and the high cooling rates typical of welding. The solute concentration during dendrite growth affects structure morphology. Greater redistribution results in finer structures, with closer dendrite arm spacing. Higher cooling rates promote rapid solidification, leading to compact dendritic growth. In such cases, solute diffusion in the liquid is limited, resulting in finer spacing patterns [42]. In commercial alloys, interdendritic spacing typically ranges from 30 to 100 µm [43].

The relative performance of the alloys in ferric chloride solution testing correlates with behavior in real environments such as seawater and highly oxidizing, low-pH, chloride containing media. Method A of the ASTM G48 standard is designed to accelerate the initiation of localized corrosion, often producing more damage than would occur in real world conditions over a similar time period [30]. After removing the specimens from the ferric chloride solution, overlay surfaces were inspected for pitting. The first inspection using a stereomicroscope revealed isolated pits on CP1, shown in Figure 12.

Specimen CP1 had an average iron content of ~19 wt.%, significantly above the recommended maximum of 5 wt.% for Inconel 625. This high Fe content can compromise corrosion resistance, as Fe tends to substitute Ni in the matrix and promote the formation of secondary phases such as TCP (Laves phase) Fe_2_Nb [44,45]. A higher magnification of the pit in Figure 13b reveals a blurry image, commonly attributed to low conductivity corrosion products adhering to the surface. Poor electrical conductivity causes surface charging under electron beam exposure, deflecting the beam due to repulsion of like charges [46].

The image more clearly confirms that the indication is indeed a pit or initiation of pitting corrosion since the affected surface shows shallow depth and size. The observed shadowing is a result of SEM surface charging, due to the presence of corrosion products with low conductivity. In the pit area, dendrites appear prominently, likely due to preferential corrosion attack along interdendritic segregation zones. The other specimens, with ~4 wt.%, ~8 wt.%, and ~12 wt.% iron content, showed no visible pitting.

In this study, microstructural analyses were carried out using SEM/EDS to identify the precipitation of secondary phases within the γ-FCC matrix of the Inconel 625 alloy. Although Inconel 625 is considered a solid solution strengthened alloy and does not require heat treatment to improve mechanical properties, its chemical composition includes a considerable amount of niobium (Nb), ranging from 3.15 wt.% to 4.15 wt.% by weight [14]. This makes the alloy susceptible to the precipitation of secondary phases such as γ″, δ, TCP phases, and carbides, especially when exposed for long periods to high temperatures (typically above 700 °C) or when there is excessive solute supersaturation in interdendritic regions, these secondary phases make the alloy more susceptible to corrosion [11,16,25,45,47].

In the analysis of Specimen CP1, a possible TCP (Laves) phase was identified precipitating in the interdendritic regions of the cellular structure, as indicated by the arrows in Figure 14. These phases appear as brighter regions in the image.

Due to the high iron content in CP1, around ~19 wt.%, the presence of the Laves phase becomes more likely [44]. A higher iron content widens the solidification temperature range of the Inconel 625 alloy, enabling a eutectic reaction involving the formation of the possible Fe_2_Nb intermetallic Laves phase. The solidification path follows this evolution [20]:

(2) $$L\to L+\gamma \to L+\gamma +Laves\to \gamma +Laves({\left(Ni,Fe\right)}_{2}(Nb,Mo))$$

The EDS-based chemical analysis of the dendritic microstructure revealed clear microscale segregation of Fe, Cr, Ni, Nb, and Mo. Partition coefficients calculated according to Equation (1) and summarized in Table 4 confirm that Nb and Mo are preferentially enriched in the interdendritic regions, where Fe and Ni are mainly concentrated in the dendritic cores. Chromium showed no significant segregation at the applied resolution. The color-coded chemical maps shown in Figure 15 further confirm these segregation trends and illustrate the redistribution of alloying elements caused by rapid solidification during welding [9,41,47].

The microsegregation of Mo and Nb observed in this study has a direct influence on the initiation of pitting corrosion in the Inconel 625 weld overlays. As shown by the EDS results and chemical maps in Figure 15, Mo and Nb are concentrated in the interdendritic regions, whereas higher Fe and Ni contents are found in the dendritic cores. This compositional heterogeneity leads to local electrochemical differences within the microstructure and favors the formation of micro-galvanic cells between interdendritic Mo/Nb-rich regions and Mo/Nb-depleted dendritic areas.

A comparable behavior was reported by Liu et al. [29] for laser-cladded Ni–Cr–Mo coatings. Those authors showed that Mo-rich interdendritic regions present higher PREN values and greater passive film stability, while Mo-depleted dendritic regions are preferential sites for pit nucleation. They also demonstrated that localized corrosion develops mainly by selective dissolution of the dendritic matrix, which is consistent with the behavior observed in the present study for specimen CP1.

In CP1, which exhibited the highest iron content (~19 wt.%), Nb and Mo segregation was associated with the precipitation of secondary Laves-type phases (Ni,Fe)_2_(Nb,Mo) in the interdendritic regions, as shown in Figure 14. The formation of these phases consumes Nb and Mo from the surrounding matrix, leading to local depletion of these elements in the dendritic regions and reducing the stability of the passive film in these areas. Similar effects associated with Laves-phase precipitation and Nb/Mo depletion in Inconel 625 overlays have been reported in previous studies [20,41,47].

Molybdenum plays an important role in localized corrosion resistance by improving both the stability and the repassivation ability of the passive film. During localized breakdown, Mo can dissolve as MoO_4_^2−^ species, which promote the regeneration of a Cr_2_O_3_-rich passive layer. In Mo- and Nb-depleted dendritic regions, this repassivation mechanism becomes less effective, resulting in higher point-defect density in the passive film and increased susceptibility to chloride-induced pit nucleation and growth [9,47].

Therefore, the occurrence of pitting in specimen CP1 is attributed not only to the high overall iron content but mainly to the combined effects of Nb and Mo microsegregation, Laves-phase precipitation, and local depletion of these elements in the dendritic matrix. This mechanism explains why specimens CP2, CP3, and CP4, with lower Fe contents and reduced segregation, did not exhibit pitting after the ASTM G48 test in this condition. The present results confirm that pit initiation in Inconel 625 weld overlays is controlled by the interaction between iron dilution, microstructural segregation, and passive film stability.

**Figure 16:**
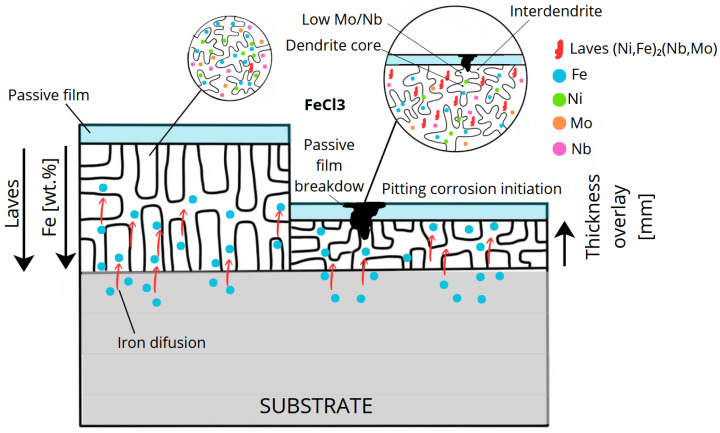
Schematic illustration of the pitting corrosion mechanism in Inconel 625 weld overlay with high Fe dilution, showing Fe diffusion from the carbon steel substrate, Nb and Mo microsegregation, Laves-type phase precipitation in the interdendritic regions, local depletion of Mo/Nb in the dendritic matrix, passive film breakdown, and pit initiation under ASTM G48 conditions.

The proposed mechanism of pitting corrosion initiation in the weld overlay with high Fe dilution is schematically illustrated in Figure 16.

The carbon steel used as the substrate for the deposition of the Inconel 625 weld overlays generally exhibits yield strength values in the range of 240–260 MPa, ultimate tensile strength between 415 and 485 MPa, and hardness of approximately 140–180 HV, depending on the material grade and its delivery condition. These values are consistent with those commonly reported for ASME Section II, Part A–SA-516 Grade 7 steels and similar carbon steels widely applied in pressure vessels and petrochemical equipment [33,47].

In contrast, the mechanical properties of the Inconel 625 weld metal are governed by the characteristics of the ERNiCrMo-3 filler metal. According to typical requirements and literature data for this consumable, the room-temperature yield strength is at least about 410 MPa, the ultimate tensile strength is approximately 760–900 MPa, and the elongation is typically higher than 30%, with hardness values commonly reported in the range of 220–260 HV [20,28,48]. These properties are mainly associated with solid-solution strengthening promoted by Nb and Mo in the nickel matrix, as well as with the refined dendritic microstructure formed during solidification of the weld metal [20].

From a design and application standpoint, the marked difference in mechanical behavior between the carbon steel substrate and the Inconel 625 cladding clearly reflects the role of each material in the clad system: the carbon steel acts as the structural component responsible for load bearing, whereas the Inconel 625 overlay provides protection against aggressive corrosive environments. This combination results in a favorable balance between mechanical strength and environmental resistance, which is essential for offshore and petrochemical applications [33].

## 5. Conclusions

Using the applied methodology, it was possible to obtain Inconel 625 weld overlay surfaces with controlled iron contents of ~4, ~8, ~12, and ~19 wt.%, enabling a systematic evaluation of the effect of Fe dilution on microstructure and corrosion behavior. All overlays exhibited dendritic microstructures with combined columnar and equiaxed morphologies, with secondary dendrite arm spacings below 10 µm, confirming the high cooling rates imposed by the GMAW process.

The corrosion tests performed according to ASTM G48 Method A demonstrated that pitting corrosion occurred exclusively in the specimen containing the highest iron content ~19 wt.%, while overlays with ~4 to ~12 wt.% Fe remained fully resistant to localized corrosion under the same testing conditions. This result establishes a critical threshold close to ~19 wt.% Fe for the onset of pitting under the experimental conditions adopted.

Microstructural and SEM/EDS analyses confirmed pronounced microsegregation of Nb and Mo during solidification, with enrichment in the interdendritic regions and relative depletion in the dendritic cores. In the CP1 overlay, this segregation was accompanied by the precipitation of Laves-type phases (Ni,Fe)_2_(Nb,Mo) in the interdendritic regions. The formation of these phases promoted local depletion of Nb and Mo in the dendritic matrix, impairing passive film stability and favoring pit initiation in these regions.

From an engineering standpoint, the results confirm that optimized multilayer buttering and controlled deposition strategies are effective in limiting Fe dilution and reducing microsegregation effects. Overlays with Fe contents up to ~12 wt.% showed stable passive behavior and no pitting under the present test conditions, indicating that corrosion-resistant Inconel 625 overlay can be reliably produced on carbon steel substrates with proper control of dilution.

Overall, this study demonstrates that the pitting corrosion resistance of Inconel 625 weld overlays is governed by the combined interaction between iron dilution, Nb/Mo microsegregation, secondary phase precipitation, and local passive film stability. These findings provide a sound metallurgical and electrochemical basis for process optimization in offshore, petrochemical, and refinery applications, contributing to improved service reliability and extended component lifetime.

The results obtained in this study demonstrate a strong potential for industrial application of the proposed Inconel 625 weld overlay strategies, particularly in components exposed to highly aggressive environments, such as offshore platforms, refineries, and petrochemical units. The identification of an iron dilution threshold associated with the onset of pitting corrosion provides a technically sound criterion for process optimization in the fabrication and repair of heat exchangers, pressure vessels, piping systems, and reactors, contributing to improved service reliability, extended component lifetime, and reduced maintenance costs.

From a scientific perspective, future research should extend the present investigation through the application of electrochemical techniques, particularly potentiodynamic polarization tests, to quantitatively assess the stability, passivation ability, and breakdown behavior of the passive film formed on the Inconel 625 overlay. Furthermore, systematic studies considering longer exposure times and a broader range of temperatures under the ASTM G48 methodology are recommended to enable a more comprehensive extrapolation of the long-term corrosion performance of the overlays under severe service conditions. A direct comparison of mechanical properties before and after corrosion testing was not performed in the present study, since mechanical characterization was beyond the defined experimental scope, which focused on microstructural features and corrosion performance. Nevertheless, future work will include post-corrosion mechanical evaluation of the clad system to assess the combined effects of iron dilution and localized corrosion on the structural integrity of the overlays.

## Figures and Tables

**Table 1:** Chemical composition of the SA-516 Grade 70 base metal and ERNiCrMo-3 filler metal (wt.%).

Elements wt.%	Fe	C	Mn	Si	Cr	Ni	Mo	Cu	S	P	Nb + Ta
SA-516 Gr 70	Rest.	0.23	1.0	0.2	0.01	0.01	0.00	-	0.004	0.013	-
ERNiCrMo-3	0.35	0.01	0.03	0.1	21.9	64.6	8.91	0.02	0.001	0.005	3.63

**Table 2:** Welding parameters used for the overlay.

Parameter	Value ^1^	Parameter	Value ^1^
Voltage [V]	30–31	Shielding gas flow rate [L/min]	14–18
Current [A]	190–210	Bead width [mm]	7
Torch travel speed [cm/min]	35	Bead height [mm]	4
Transfer mode	Globular	Wire feed speed [m/min]	31
Heat input [kJ/mm]	1.2	Torch angle [°]	91
Maximum temperature [°C]	145	Wire diameter [mm]	1.2

^1^ Values correspond to the average conditions used during the overlay welding.

**Table 3:** Chemical composition (wt.%) of the Inconel 625 weld overlays (CP1–CP4) determined by XRF analysis, and nominal chemical composition of the ERNiCrMo-3 filler metal according to ASME Section II, Part C–SFA 5.14.

Specimen		Fe ^1^ [%]	Cr ^1^ [%]	Ni ^1^ [%]	Mo ^1^ [%]	Ti ^1^ [%]	Nb ^1^ [%]
ERNiCrMo-3		5.00	20.00–23.00	Min. 58.00	8.00–10.00	0.40	3.15–4.15
CP1	Found Deviation	19.16 +14.16	19.23 −0.77	51.2 −6.80	7.40 −0.60	0.20 -	2.77 −0.38
CP2	Found Deviation	7.47 +2.47	21.63	59.50	8.57	0.20	3.23
			-	-	-	-	-
CP3	Found Deviation	12.06 +7.06	20.60 -	55.83 −2.17	8.03 -	0.20 -	3.03 −0.12
CP4	Found Deviation	3.83 -	21.16 -	61.50 -	8.87 -	0.20 -	3.33 -

^1^ Error ± 0.5%.

**Table 4:** Partition coefficients focused on Laves-related elements were calculated according to Equation (1).

Specimen	Cr (K) ^1^	Cr (seg.) ^1^	Mo (K) ^1^	Mo (seg.) ^1^	Nb (K) ^1^	Nb (seg.) ^1^
CP1	1.10	Core ↑	0.82	Inter ↑	0.36	Inter ↑
CP2	1.08	Core ↑	0.86	Inter ↑	0.47	Inter ↑
CP3	1.05	≈	0.90	Inter ↑	0.60	Inter ↑
CP4	1.02	≈	0.95	≈	0.78	Inter ↑

^1^ (i) K < 1 indicates interdendritic enrichment (Inter ↑), consistent with Nb/Mo segregation that promotes Laves (Ni,Fe)_2_(Nb,Mo); (ii) K ≈ 1 indicates minimal segregation; (iii) K > 1 indicates core enrichment (Core ↑). Rearranged per reviewer to highlight Cr, Mo and Nb trends relevant to Laves formation.

## Data Availability

The original contributions presented in this study are included in the article. Further inquiries can be directed to the corresponding author.

## Abbreviations

The following abbreviations are used in this manuscript:

Abbreviations	
API RP 582	Welding Guidelines for the Chemical, Oil, and Gas Industries
ASME	American Society of Mechanical Engineers
ASTM G48	Standard Test Method for Pitting and Crevice Corrosion Using Ferric Chloride
CP	Test specimen
EDS	Energy Dispersive Spectroscopy
ERNiCrMo-3	Nickel-based alloy welding filler metal (Inconel 625 classification)
FCC	Face-Centered Cubic
GMAW	Gas Metal Arc Welding
GTAW	Gas Tungsten Arc Welding
HAZ	Heat-Affected Zone
NORSOK M-601	Materials and Welding Requirements for Offshore Structures
PREN	Pitting Resistance Equivalent Number
SEM	Scanning Electron Microscopy
XRF	X-ray Fluorescence
Symbols and Variables	
HV	Vickers Hardness
K	Partition coefficient
Cs	Element concentration in the dendrite core (solid phase)
Cl	Element concentration in the interdendritic region (liquid phase)
γ	Gamma nickel solid solution phase
t	Time [h]
T	Temperature [°C]
i	Current [A]
wt.%	Weight percentage
µm	Micrometer
mm	Millimeter
V	Voltage (Volts)
A	Electric current (Ampers)
L/min	Gas flow rate
kJ/mm	Heat input
Chemical Compounds and Phases	
FeCl_3_	Ferric chloride
HCl	Hydrochloric acid
HNO_3_	Nitric acid
γ	Gamma nickel solid solution (matrix phase)
γ″	Gamma double-prime strengthening phase (Ni_3_Nb)
δ	Delta phase (orthorhombic Ni_3_Nb)
TCP	Topologically close-packed intermetallic phases
Laves	Intermetallic Laves phase (Ni,Fe)_2_(Nb,Mo)
Carbides	Secondary carbide precipitates
